# Investigation of Vibration-Induced Transport of Newtonian and Non-Newtonian Fluids in Porous Media Using Lattice Boltzmann Method

**DOI:** 10.3390/bioengineering13010036

**Published:** 2025-12-28

**Authors:** Soon Wook Kwon, Hee Min Lee, Hyun Cheol Yeom, Chang Sup Oh, Joon Sang Lee

**Affiliations:** 1 Division of Quantum Computing, Department of Mechanical Engineering, Yonsei University, Seoul 03722, Republic of Korea; erickwon99@gmail.com (S.W.K.); heemin005@gmail.com (H.M.L.); 2 Medihub Inc., Gunpo-si 15808, Republic of Korea; maro@medihub.co.kr (H.C.Y.); csoh@medihub.co.kr (C.S.O.); 3 Center for Precision Medicine Platform Based on Smart Hemo-Dynamic Index (SHDI), Seoul 03722, Republic of Korea

**Keywords:** vibration-assisted injection, non-Newtonian rheology, porous media, lattice Boltzmann method, drug uptake

## Abstract

Pain and variable uptake remain practical barriers to needle-based delivery. Device-level vibration has emerged as a simple strategy for improving tolerability and dispersion, but its fluid-mechanical basis remains incomplete. Using a lattice Boltzmann model with a porous-media skin surrogate, we applied time-periodic inlet pressures at 0%, 16.6% ($\Delta P_{1}$), and 35.1% ($\Delta P_{2}$) amplitudes to Newtonian, model shear-thinning, and clinically measured protein formulations. We quantified the wall shear stress, wetted area, dispersion length, and pressure cost over one cycle. Vibration increased the normalized wetted area by 10.6% for Newtonian flow and by 15.9% and 21.3% for the non-Newtonian cases at $\Delta P_{1}$ and $\Delta P_{2}$, respectively, while advancing the penetration front and lateral dispersion. The one-cycle pressure cost per wetted area decreased by 3.9% for Newtonian flow and by 5.96% and 7.80% for non-Newtonian flows. For shear-thinning fluids, the wall-shear history was reshaped, with a brief early amplification and late-phase mean reductions of 10.3% and 13.3% at $\Delta P_{1}$ and $\Delta P_{2}$. These results establish a fluid-mechanical mechanism linking clinically relevant vibration amplitudes to reduced sustained shear exposure, deeper and broader depot formation, and improved conditions for drug uptake.

## 1. Introduction

The fear of needles is common and clinically consequential. A systematic review and meta-analysis has shown that needle fear affects most children, 20–50% of adolescents, and 20–30% of young adults, with measurable effects on healthcare avoidance [1]. Pain is the principal driver of the fear of needles and its avoidance. In a large international survey of adults, respondents most frequently cited general anxiety and pain as reasons for needle fear and reported avoiding blood draws, donations, or vaccinations as a result [2]. The longitudinal and guideline literature further indicates that painful or poorly managed needle procedures in childhood contribute to needle fear that can persist into adulthood and undermine adherence, underscoring the importance of directly reducing injection pain [3,4,5].

Vibration-assisted injection has emerged as a practical option to reduce injection-related pain. Several clinical studies have reported lower pain scores across procedures and age groups, including dermatology and aesthetic injections [6,7], dentistry [8], and perioperative or pediatric contexts [9]. Although effect sizes vary by protocol and setting, aggregate evidence indicates that adding vibrations can considerably attenuate perceived pain during injections.

Many studies have examined the influence of vibration-assisted injections on perceived pain reduction, and two explanations have been proposed. First, vibration at the needle–tissue interface decreases insertion and frictional forces, which correlates with reduced puncture-related pain; benchtop and in-tissue studies have shown substantial decreases in peak insertion force when axial oscillation is applied [10,11,12]. Second, in transdermal and microneedle delivery, externally applied oscillations can facilitate microchannel formation and transiently increase cutaneous permeability, thereby facilitating intradermal access and drug transport [13,14].

By contrast, we consider a third mechanism to be critical: the fluid mechanics of the injection process. In vibration-assisted injection, oscillatory forces can reorganize flow in tissue-like porous matrices; notably, for non-Newtonian injectates, the apparent viscosity can change under oscillation, altering pressure and shear fields that drive nociceptor activation [15,16]. This pathway is particularly crucial because many clinically used injectates are non-Newtonian fluids, such as hyaluronic acid fillers and viscosupplements, which exhibit shear-thinning with amplitude- and rate-dependent responses in oscillatory tests. This implies that vibration can reorganize their flow differently from Newtonian solutions [17,18,19,20]. Therefore, we hypothesized that vibration-induced, rheology-dependent flow reorganization could reduce nociceptor-relevant mechanical stimuli by decreasing peak infusion pressures and redistributing wall shear. This is consistent with human data linking higher infusion pressures to greater reported pain during intradermal injection [21].

**Figure 1:**
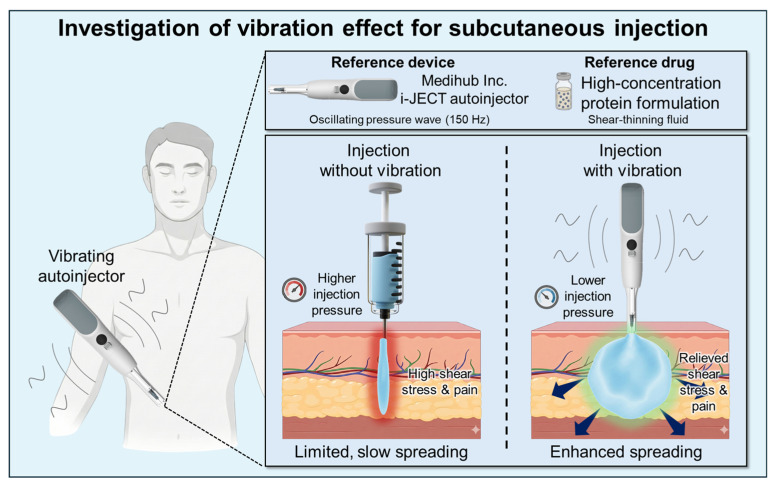
Overview of vibration-assisted injection. The syringe vibrates continuously during injection while the skin remains stationary; the zoomed view compares dispersion with and without vibration, highlighting enhanced lateral spreading.

Despite the progress in oscillatory transport in porous structures and rheology, previous studies primarily characterized the flow and permeability rather than connecting vibration-induced hydrodynamic fields to pain-relevant mechanical cues in skin-like media. To the best of our knowledge, no study had directly linked these vibration-driven flow reorganizations in a tissue-porous surrogate to quantitative proxies of the nociceptor drive, such as local pressure transients, wall shear stresses, and the inlet pressure required to sustain a clinically relevant flow. This link is important because human intradermal experiments have associated higher infusion pressures with greater reported pain [21]. Throughout this paper, vibration refers to oscillations applied to the syringe system during injection whereas the skin remains stationary. Figure 1 summarizes the assumed scenario and study focus by contrasting dispersion with and without vibration in a zoomed skin cutaway, motivating the subsequent analyses of wetting, wall shear, and pressure.

In this study, we addressed this gap by analyzing vibration-assisted injection from a fluid-mechanical perspective that was explicitly anchored to pain-relevant metrics. The skin was idealized as a porous medium using a cylindrical needle, and syringe vibration was imposed as a time-periodic inlet pressure. Under identical geometry and boundary conditions, we compared Newtonian and non-Newtonian injectates and quantified three mechanistic readouts related to nociception: spatiotemporal pressure fields, wall shear stress distributions, and the wetting area at the tissue–fluid interface. We also measured the inlet pressure required to deliver the same volumetric flow, which provided a direct surrogate for the perceived injection force.

The specific contributions of our study are as follows. (1) We provided a controlled comparison between Newtonian and non-Newtonian injectates under identical pressure-wave forcing in a porous-media skin surrogate, isolating the role of rheology in vibration-induced flow reorganization. (2) We mapped how the forcing amplitude reshapes pain-relevant fields by jointly analyzing the peak and integrated pressure, wall shear stress, wetting area, and pressure cost of achieving a fixed flow, thereby translating vibration-induced transport phenomena into nociceptor-relevant mechanical stimuli. (3) We proposed a mechanistic rationale for pain reduction during vibration-assisted injection. The rheology-dependent redistribution of pressure and shear reduces peak stimuli while maintaining delivery, offering a fluid-mechanical basis for clinical observations of reduced pain with vibration.

## 2. Numerical Methods

A pseudopotential lattice Boltzmann method (LBM) model was employed in this study to simulate the multiphase flow within porous media. The LBM is a computational fluid dynamics approach that recovers macroscopic flow properties through iterative collision and streaming processes of the distribution function f. Because of its methodological advantages, the LBM is particularly suitable for simulating flows through porous structures [22]. The evolution equation of the pseudopotential LBM is expressed as

(1) $$f_{i}\left(x+e_{i}\Delta t,t+\Delta t\right)-f_{i}\left(x,t\right)=-\frac{1}{\tau }\left[f_{i}\left(x,t\right)-f_{i}^{eq}\left(x,t\right)\right]+\Delta f_{i}\left(x,t\right),$$

where x denotes the position vector, t is the time, Δt is the time step, $e_{i}$ represents the lattice velocity, and τ is the relaxation time that determines the fluid viscosity, μ. The equilibrium function, $f_{i}^{eq}$ can be calculated:

(2) $$f_{i}^{eq}=\omega_{i}\rho \left[1+\frac{e_{i}\cdot u}{c_{s}^{2}}+\frac{{\left(e_{i}\cdot u\right)}^{2}}{2c_{s}^{4}}-\frac{{\left|u\right|}^{2}}{2c_{s}^{2}}\right],$$

where $\omega_{i}$ is the weighting factor and $c_{s}$ is the lattice sound speed. ρ and u are the macroscopic density and velocity, respectively.

The forcing term Δf is used to account for the external force acting on the fluid, and it is defined as follows [23].

(3) $$\Delta f_{i}\left(x,t\right)=f_{i}^{eq}\left(\rho ,u+\Delta u\right)-f_{i}^{eq}\left(\rho ,u\right),$$

where Δf is the difference between equilibrium distribution functions evaluated with different velocity values. The intermediate velocity Δu is induced by the external force, and Δu=FΔt/ρ. The macroscopic density, velocity, and viscosity were determined using the distribution functions obtained above.

(4) $$\rho =\sum_{i}f_{i},\;u=\sum_{i}f_{i}e_{i},\;\mu =\rho c_{s}^{2}\left(\tau -0.5\right),$$

Next, an interaction force $F_{int}$ must be applied to induce phase separation within the fluid to model the two immiscible phases.

(5) $$F_{int}=-\frac{3c_{0}G}{c^{2}\Delta t}\left[\beta \psi \left(x\right)\sum_{i}\omega_{i}\psi \left(x+e_{i}\Delta t\right)e_{i}+0.5\left(1-\beta \right)\sum_{i}\omega_{i}\psi^{2}\left(x+e_{i}\Delta t\right)e_{i}\right],$$

Here, β, $c_{0}$, and G are constants representing the interparticle interactions. In this study, the parameters were set to β=1.16, $c_{0}=6.0$, and G=0.5, as suggested by a previous study [24] for achieving stable simulations. ψ denotes the effective mass and is defined as a function of pressure p and density ρ, as follows:

(6) $$\psi =\sqrt{\frac{2\left(p-\rho c_{s}^{2}\right)}{c_{0}G}}.$$

The Peng–Robinson equation of state was employed to calculate the pressure.

(7) $$p=\frac{RT\rho }{1-b\rho }-\frac{a\rho^{2}\varepsilon \left(T\right)}{1+2b\rho -b^{2}\rho^{2}},$$

where T and R are the temperature and the ideal gas constant, respectively, and ε is the acentric factor. The constants, a and b, are defined based on the critical temperature and pressure, respectively. When the critical temperature and pressure were set to $T_{c}=0.0729$, $p_{c}=0.0596$, and R=1 [25], the corresponding values of a and b were a=2/49 and b=2/21. The density ratio between the two phases was determined by the temperature; in this study, $T=0.85T_{c}$ was applied.

The Cross model [26] was applied to the viscous term to model a shear-thinning non-Newtonian fluid in which the viscosity decreases with an increasing shear rate.

(8) $$\mu =\mu_{\infty }+\frac{\mu_{0}-\mu_{\infty }}{1+{\left(k\dot{\gamma }\right)}^{n}},$$

where $\mu_{0}$ is the zero-shear viscosity, $\mu_{\infty }$ is the infinite-shear viscosity, $\dot{\gamma }$ is the local shear rate, and k is the time constant. When the power-law index n satisfies n>0, the fluid exhibits shear-thinning behavior. The symmetric strain rate tensor $D_{\alpha \beta }$ must first be calculated to determine the local shear rate.

(9) $$D_{\alpha \beta }=\frac{1}{2}\left(\frac{\partial u_{\beta }}{\partial x_{\alpha }}+\frac{\partial u_{\alpha }}{\partial x_{\beta }}\right).$$

The local shear rate computed from $D_{\alpha \beta }$ is expressed by

(10) $$\dot{\gamma }=\sqrt{2D_{\alpha \beta }D_{\alpha \beta }}.$$

The variation in viscosity with the shear rate can be determined using Equation (8). Consequently, according to Equation (4), the relaxation time also varies with the modified viscosity.

## 3. Results

### 3.1. Simulation Setup

**Figure 2:**
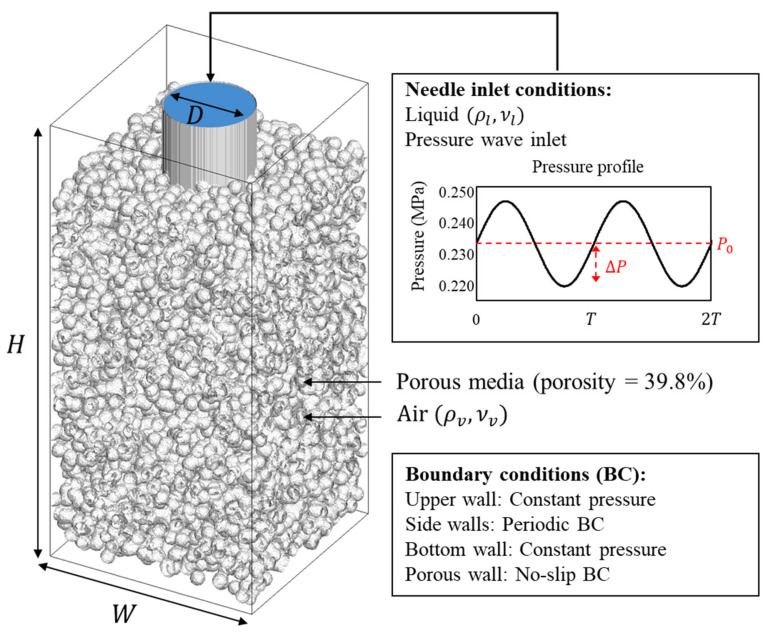
Schematic illustration of simulation domain.

A schematic of the simulation domain is shown in Figure 2. We model a subcutaneous injection: the needle traverses the skin and the tip resides in the subcutis at a surface-to-tip depth $d_{tip}$, where $d_{skin}$ denotes the surface-to-skin–fat boundary and $h_{insert}$ the local penetration below that boundary within the computational window. In the baseline, we set $d_{tip}\approx \;3.0\;mm$ and $h_{insert}=\;0.20\;mm$, as listed in Table 1.

The computational domain is a cropped, rigid, isotropic porous media window surrounding the stationary tip; puncture and tissue deformation are not simulated because the objective is to isolate how syringe vibration reorganizes fluid transport while the skin remains stationary. The needle is represented as a straight hollow cylinder terminating in a flat circular outlet into the porous media. This idealization preserves the effective hydraulic area and axial alignment while removing bevel details that are not central to the mechanism.

The needle corresponds to a clinical 32.5 G device with an inner diameter of 0.11 mm. The porous matrix is generated by randomly placing solid inclusions until a target porosity is reached. To ensure a controlled comparison and prevent geometric artifacts, we utilize a single representative realization with a volume fraction of void ε=0.398 and a mean pore size of approximately 21.3 μm. This porosity level is chosen to represent a skin-like, highly permeable scaffold with interconnected pathways for transport, consistent with biomaterial guidance that porosity at or above about 40% supports realistic permeability–mechanics trade-offs in skin-relevant constructs [27]. The chosen porosity, with pore sizes of 21.3 μm, yields permeabilities in the 10^−12^–10^−11^ m^2^ range that are consistent with subcutaneous tissue in poromechanics coupled injection models.

The inlet applies a prescribed pressure waveform; the outlet is held at constant pressure. The inlet pressure uses a baseline value of $P_{0}=0.232\;\mathrm{MPa}$. Three driving profiles are defined and will be used consistently throughout the analysis: a steady profile at $P_{0}$, a sinusoidal profile with amplitude $\Delta P_{1}=16.6\%$ of $P_{0}$, and a sinusoidal profile with amplitude $\Delta P_{2}=35.1\%$ of $P_{0}$. The corresponding waveforms are $P(t)=P_{0},P(t)=P_{0}[1+(\Delta P_{1}/P_{0})sin(2\pi t/T)]$ and $,P(t)=P_{0}[1+(\Delta P_{2}/P_{0})sin(2\pi t/T)]$. The period T is set by a 150 Hz cycle, so $T=1/150\;s\approx 6.667\times 10^{-3}\;s$. The inlet boundary profile, including the prescribed injection velocity and vibration waveform, was derived from data for the commercial I-ject autoinjector (MEDIHUB Inc.). All time-resolved results are presented in nondimensional form with $t^{*}=T$. Also, the simulation domain size and fluid properties are shown in Table 1.

### 3.2. Viscosity Model Validation

**Figure 3:**
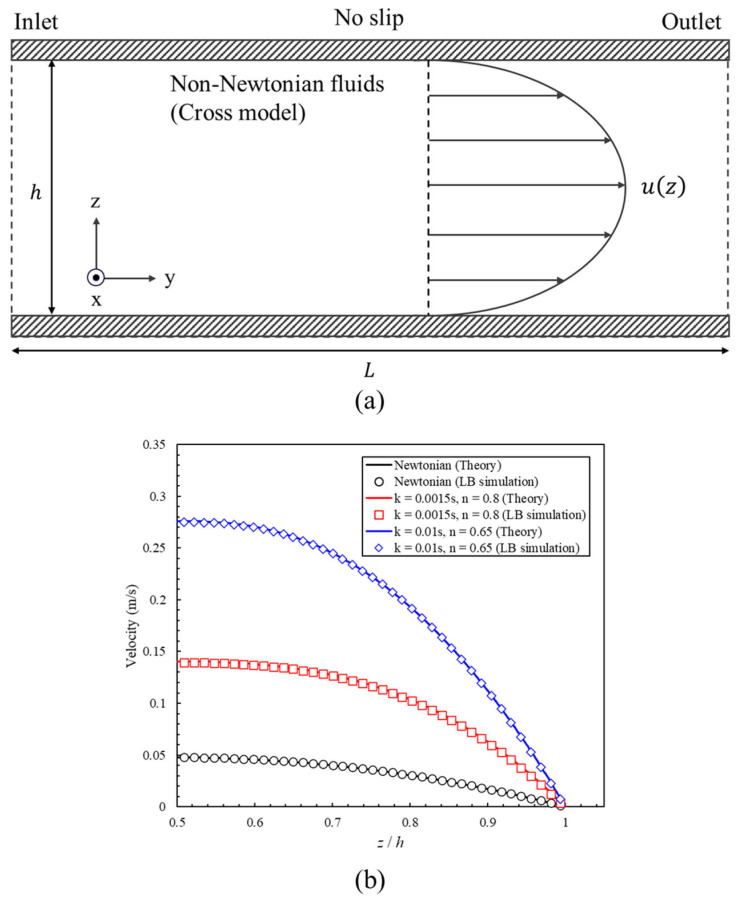
Non-Newtonian model validation in pressure-driven Poiseuille flow. (**a**) Schematic of simulation domain. (**b**) Validation results. The lines represent theoretical solutions and the symbols represent LBM simulations.

We validated the non-Newtonian viscosity model and the proposed LBM implementation using a steady pressure-driven Poiseuille flow between two parallel plates. Figure 3a illustrates the schematic of the validation case domain, where the domain height h and length L were set to 80 and 240 lattice units, respectively. The top and bottom surfaces were defined as walls with a no-slip boundary condition while the inlet and outlet employed constant pressure boundary conditions. When the fluid followed the non-Newtonian Cross model described in Equation (8), the velocity profile in Poiseuille flow was expressed as follows.

(11) $$u\left(z\right)=\frac{h}{\tau_{w}}\int_{\dot{\gamma }\left(z\right)}^{{\dot{\gamma }}_{w}}\dot{\gamma }\left[\mu_{\infty }+\left(\mu_{0}-\mu_{\infty }\right)\frac{1+{\left(k\dot{\gamma }\right)}^{n}\left(1-n\right)}{{\left[1+{\left(k\dot{\gamma }\right)}^{n}\right]}^{2}}\right]d\dot{\gamma },$$

where $\tau_{w}$ denotes the wall shear stress and ${\dot{\gamma }}_{w}$ represents the wall shear rate.

The velocity profiles of one Newtonian fluid and two non-Newtonian fluids listed in Table 2 were compared with theoretical solutions. The LBM results were consistent with the reference solutions over the entire cross-section for all three property sets, which can be seen in Figure 3b. In the Newtonian case, the simulated profile was visually indistinguishable from the analytical parabola, confirming the correctness of the viscosity and boundary condition treatments. In the two Cross cases, the simulations replicated the expected shear-thinning behavior: as k increased and n decreased, the centerline velocity increased, and the profile became fuller under the same pressure gradient. These results verify that the viscosity update and shear rate evaluation based on the proposed LBM solver are accurate and stable.

### 3.3. Effect of Vibration on Wetting Area

We began by analyzing the wetting area A(t), defined as the portion of the porous wall in direct contact with the liquid at time t. In the simulation, a wall surface node was considered as wetted if any of its adjacent lattice nodes contained liquid, and the instantaneous area was computed as $A(t)=N_{wet}(t)dx^{2}$, where $N_{wet}(t)$ was the number of wetted wall nodes and dx was the lattice spacing. For comparison across cases, we report the normalized metric $A/A^{*}$, where $A^{*}$ is the total accessible wall area within the field of view.

**Figure 4:**
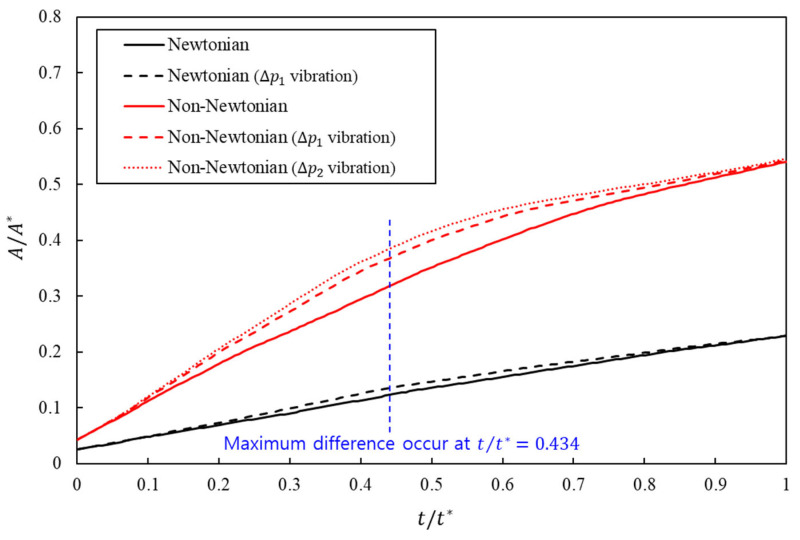
Time evolution of normalized wetting area $A/A^{*}$ versus nondimensional time $t/t^{*}$ for Newtonian and non-Newtonian fluids with and without inlet-pressure vibration. The black and red colors represent the results for the Newtonian and non-Newtonian fluids, respectively. The dashed line indicates that inlet-pressure vibration is applied.

**Figure 5:**
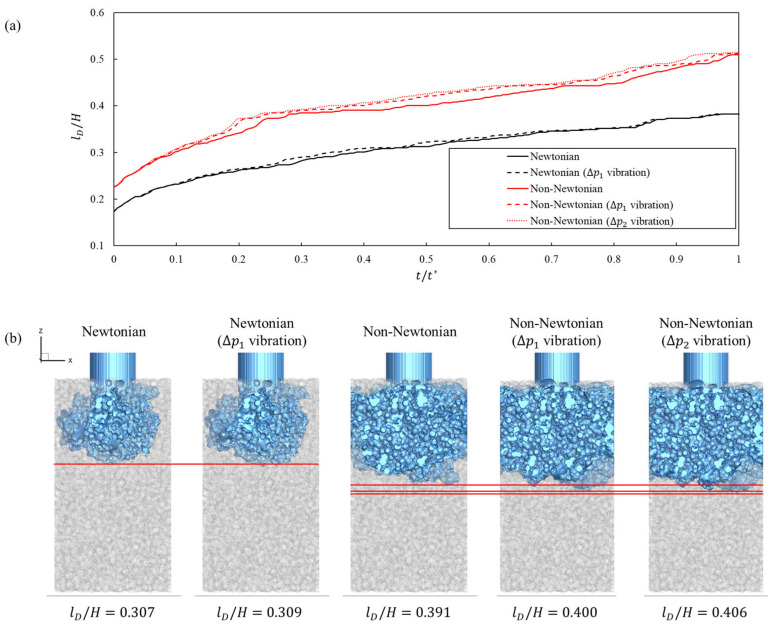
(**a**) Quantitative plot of fluid dispersion length and (**b**) qualitative plot of fluid dispersion length for each fluid type and vibration amplitude at $t/t^{*}=0.434$. The red line has been added to help compare dispersion lengths. The dispersion length $l_{D}$ is nondimensionalized using the domain height H.

Figure 4 shows the evolution of $A/A^{*}$ over nondimensional time $t/t^{*}$ for Newtonian and Cross fluids with and without inlet-pressure vibration. The curves separated early and reached their largest gap at $t/t^{*}=0.434$, indicating that vibration accelerates the spread of liquid contact along the wall. At this instant, the wetting metric exceeds the corresponding no-vibration baseline by 10.61% for the Newtonian fluid at a $\Delta P_{1}$ amplitude. The non-Newtonian formulation exhibited larger gains, with 15.85% at a $\Delta P_{1}$ amplitude and 21.34% at a $\Delta P_{2}$ amplitude. These values are listed in Table 3. Increasing the amplitude from $\Delta P_{1}$ to $\Delta P_{2}$ amplitude results in an increase in the non-Newtonian gain by only 5.49%. Consistent with the wetting trends, Figure 5a shows that, at the same nondimensional time, the normalized dispersion length is always greater when inlet-pressure vibration is applied, with the largest enhancement for the shear-thinning cases. For a given dispersion length, the corresponding time is reduced under vibration, indicating that oscillatory forcing advances the penetration front and accelerates the onset of lateral spreading in the porous window. This implies a sublinear response consistent with shear-thinning behavior in which further increases in local shear result in diminishing reductions in apparent viscosity (Figure 5b).

The wetting results were interpreted in the context of subcutaneous and intradermal deliveries. After injection, the drug forms a local depot that spreads through the extracellular matrix; the extent of spreading increases the contacted tissue area and is associated with enhanced absorption and bioavailability [28]. Reviews and clinical studies have reported that enhancing dispersion in subcutaneous tissue increases uptake; for example, using hyaluronidase to transiently open the hyaluronan network enlarges the dispersion area and accelerates the absorption of co-administered drugs and fluids [29,30,31]. Similarly, imaging and pharmacokinetic analyses of insulin injections showed that the lateral spreading of the depot within the subcutaneous layer correlates with increased absorption dynamics [32,33]. 

Consistent with these observations, recent high-fidelity computational models of subcutaneous injection that couple poromechanics with multi-network transport also show that the lateral widening of the depot accompanies faster uptake, reinforcing this link [34,35]. The larger wetting area in our simulations indicates that a larger portion of the porous wall is in contact with the liquid, which is a proxy for a larger contacted tissue interface in vivo. Therefore, the observed increases in $A/A^{*}$ under vibration are not only hydrodynamic differences but also suggest a practical route to improve injection performance by expanding contact and promoting efficient uptake without changing the drug or formulation.

### 3.4. Effect of Vibration on Wall Shear Stress

**Figure 6:**
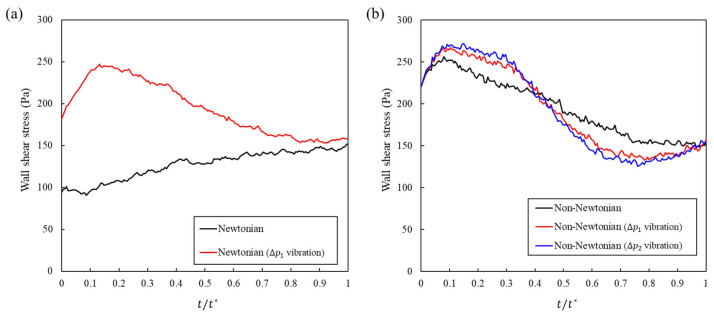
Wall shear stress (WSS) over one pressure vibration cycle for Newtonian and non-Newtonian formulations, with and without vibration. (**a**) Newtonian: the curve with vibration remains above the no-vibration reference and reaches its peak earlier, indicating globally higher near-wall shear. (**b**) Non-Newtonian: vibration raises WSS in the early to middle interval and advances the peak, followed by a modest decrease below the reference in the late interval, consistent with shear-induced viscosity reduction and saturation; the late-phase unloading represents a clinically favorable redistribution of mechanical load.

We analyzed the wall shear stress τ(t) over a full pressure cycle, with time normalized by the period $t^{*}$. In the Newtonian formulation, vibration increases τ(t) throughout the cycle and causes the peak to be attained earlier, which indicates the global amplification of near-wall shear and faster dynamics, as shown in Figure 6a. This increment is reflected in Table 4. The late-phase mean over the final 60% of the cycle increases from 142.64 Pa to 162.02 Pa. The 95th percentile increases from 147.08 Pa to 243.98 Pa. Because the shear components of the mechanical load can activate cutaneous nociceptors and drive rapid mechanical pain signaling, a higher and more sustained wall shear stress (WSS) profile is expected to worsen pain [36].

The shear-thinning formulation responded differently, and the difference was clinically favorable. Vibration front-loads τ(t) early and unloads it later, as shown in Figure 6b. In Table 4, the late-phase mean decreases from 158.62 Pa to 142.22 Pa with a $\Delta P_{1}$ vibration amplitude and decreases to 137.53 Pa with a $\Delta P_{2}$ vibration amplitude. These correspond to reductions of approximately 10.3% and 13.3% relative to the no-vibration case. The peak increased in size and was attained earlier, and the 95th percentile increased to 262.98 and 267.56 Pa. Because nociceptors are driven by shear, and sustained shear is undesirable for pain, this temporal redistribution, that is, an earlier and larger response followed by a lower late-phase average, matches the pain-mitigating WSS profile without changing the drug.

Across the datasets, these results provide a clear design choice. The application of vibration to a shear-thinning formulation concentrates shear when the apparent viscosity is the most labile and then reduces sustained exposure later in the cycle.

**Figure 7:**
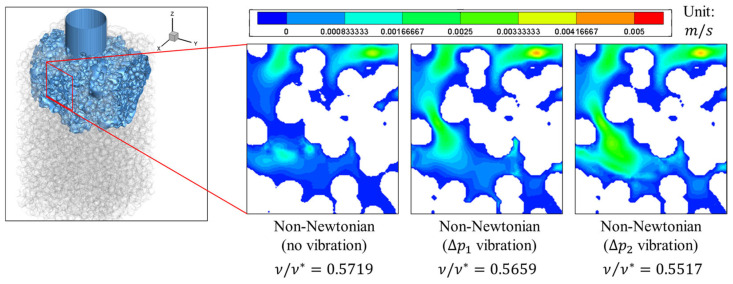
Instantaneous velocity magnitude field at the nondimensional time $t/t^{*}=0.149$, corresponding to the peak wall shear τ. Under vibration, the near-wall flow accelerates in an amplitude-dependent manner: the $\Delta P_{2}$ amplitude case exhibits broader high-speed corridors and faster wall-adjacent streams than the $\Delta P_{1}$ amplitude case, yielding a larger τ at this phase. The color scale denotes ∣u∣; all cases share the same domain and visualization parameters. *Capture location:* analysis window of width 0.10W and height 0.07W centered at (x/W, y/W)=(0.64, 0.42).

At the nondimensional time $t/t^{*}=0.149$, when the wall shear attains its peak, the instantaneous velocity fields in Figure 7 reveal an amplitude-dependent acceleration of the near-wall flow. The $\Delta P_{2}$ amplitude case forms faster pore-scale streams and wider high-speed corridors adjacent to the solid boundaries, steepening local velocity gradients and increasing the wall shear, τ. By comparison, $\Delta P_{1}$ amplitude yields only a modest speed-up with thinner high-speed streaks, consistent with a lower increase in τ. These patterns indicate that increased oscillatory forcing promotes advective penetration through preferential throats and expands the footprint of the rapid flow next to the wall, which, in turn, increases the instantaneous shear at the solid–liquid interface.

Mechanistically, the non-Newtonian response amplified these amplitude effects under oscillatory pressure forcing. A higher inlet-pressure amplitude transiently increases the local shear rate and decreases the effective resistance to near-wall motion in the non-Newtonian fluid, yielding a nonlinear gain in velocity for the $\Delta P_{2}$ amplitude case and an earlier, larger peak in τ at $t/t^{*}=0.149$. The $\Delta P_{1}$ case exhibits a weaker rate-dependent reduction in resistance and correspondingly slower near-wall motion. Together with the one-cycle statistics, the snapshots in Figure 7 support the interpretation that increasing the vibration amplitude concentrates shear early in the cycle by accelerating wall-adjacent streams while preserving the late-phase unloading behavior characteristics of the non-Newtonian formulations.

### 3.5. Effect of Vibration on Pressure

We define dP/dA as the pressure required per unit wetted area at time t (units: ${Pa\cdot m}^{-2}$). This answers a direct question regarding the injection design: for the same wetting extent, what amount of pressure is required by the system. To compare conditions over one full cycle, we also show the one-cycle integral of dP/dA (units: ${Pa\cdot m}^{-2}$·cycle), which represents the total pressure cost to build the wetted interface.

**Figure 8:**
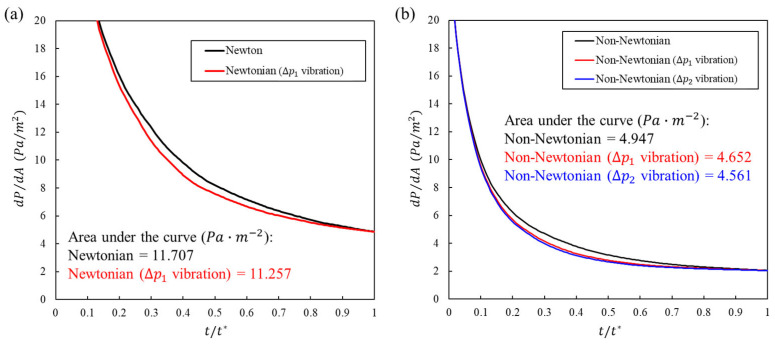
Pressure per wetted area over one cycle. (**a**) Newtonian: dP/dA with vibration is lower than baseline over most of the cycle. The panel label includes the one-cycle integral and its percent reduction relative to the baseline. (**b**) Non-Newtonian: vibration further decreases dP/dA; the annotated integrals show 5.96% and 7.80% decreases for $\Delta P_{1}$ and $\Delta P_{2}$ amplitude vibration, respectively. Lower values indicate less pressure required to achieve the same wetting, which is desirable for tolerability.

Figure 8 depicts dP/dA and annotates, inside each graph, the one-cycle integral and the percent change from the no-vibration baseline. In Figure 8a (Newtonian), the vibration curve sits below the baseline throughout most of the cycle. The one-cycle integral decreases from 11.707 to 11.257 ${Pa\cdot m}^{-2}$·cycle, a 3.9% reduction. The cycle mean corroborates this drop, from 11.640 to 11.193 ${Pa\cdot m}^{-2}$ (3.8% lower). Therefore, for the same wetting trajectory, vibration achieves the target contact using less total pressure.

In Figure 8b (non-Newtonian), the benefit is more and scales with the amplitude. The integral decreases from 4.947 to 4.652 ${Pa\cdot m}^{-2}$·cycle with a $\Delta P_{1}$ amplitude (5.96% reduction) and to 4.561 ${Pa\cdot m}^{-2}$·cycle with a $\Delta P_{2}$ amplitude (7.80% reduction). The cycle mean exhibited the same trend, from 4.919 to 4.625 and 4.535 ${Pa\cdot m}^{-2}$ (5.98% and 7.79% lower, respectively). These results indicate a clear pressure-efficiency gain from vibration, which was most pronounced for the non-Newtonian formulation.

To interpret these stress histories in a pain-relevant context, we did not introduce a new calibrated nociceptor model. Instead, we treated two mechanically meaningful quantities as surrogate drivers of nociceptor activation: the late-phase mean wall shear stress, which reflects sustained shear exposure at the tissue–fluid interface, and the cycle-integrated pressure per wetted area, which reflects the pressure cost required to achieve a given fluid–tissue contact. Experimental work has shown that shear stress can itself activate mechanosensitive nociceptors and contribute to mechanical pain signaling [36], and human microneedle injection studies have reported that higher infusion pressures are associated with higher perceived pain [21].

In dimensional terms, any mechanical drive acting on a sparse population of subcutaneous endings is expected to increase with the local density of those endings and with sustained near-wall shear and pressure per unit contact area. We therefore interpret the mechanical nociceptor drive in this study as scaling with receptor density multiplied by a weighted combination of late-phase mean wall shear stress and pressure per wetted area, and we focus on how superimposed vibration reduces these surrogates under identical geometry and delivery conditions. In the shear-thinning fundamental cases, vibration lowered late-phase mean wall shear stress by approximately 10 to 13% while also reducing the one-cycle pressure-per-area integral by about 6 to 8%, indicating a substantial unloading of sustained mechanical drive even as the wetted region expanded.

### 3.6. Case Studies with Clinically Measured Shear-Thinning Protein Formulations

To test whether the vibration-induced transport mechanisms identified in Section 3.3, Section 3.4 and Section 3.5 remain valid for a realistic injectable drug, we carried out an additional case study based on experimentally measured properties of high-concentration protein formulations reported by Marschall et al. [37]. In that study, monoclonal IgG1 antibody (mAb) and lysozyme (Lys) were each formulated with trehalose (Tre) as an excipient; we use “mAb:Tre 70:30” and “Lys:Tre 70:30” to denote mixtures in which the mass ratio of protein (mAb or Lys) to trehalose is 70:30, with a total solids (ts) content of 7.5%. The protein concentration, denoted $c_{prot}$, refers to the mass of protein per unit volume and was fixed at 280 mg/mL in the high-concentration formulations. For the lysozyme-based system, an aqueous Lys:Tre 70:30 solution in histidine buffer at $c_{prot}$ = 280 mg/mL exhibited an apparent viscosity of $\eta_{solution}\;=\;5.1\;mPa\cdot s$ at injection-relevant shear rates. For the monoclonal antibody system, mAb:Tre 70:30 powder suspensions were prepared in the semifluorinated alkane vehicle perfluorobutylpentane (F4H5), and the suspension at $c_{prot}$ = 280 mg/mL showed a substantially lower viscosity than the corresponding aqueous mAb solution, with an apparent viscosity of $\eta_{F4H5}\;=\;9.2\;mPa\cdot s$. In what follows, we use these two measured values as representative low- and high-viscosity cases within the range of injectable high-concentration protein formulations, and for brevity refer to them as the “solution” and the “F4H5”, respectively.

In our lattice Boltzmann framework, both cases were represented by the same shear-thinning Cross constitutive law, characterized by $\mu_{\infty }\;=\;1.52\;mPa\cdot s$, k = 0.00554, and n = 1.4. The only parameter that differed between the two cases was the zero-shear viscosity $\mu_{0}$, which was set to 5.1 mPa·s for the solution case and 9.2 mpa·s for the F4H5. With this choice, both formulations shared an identical shear-thinning curve shape determined by $\mu_{\infty }$, k, and n while their overall viscosity level was tuned by $\mu_{0}$ so that the apparent viscosity at injection-relevant shear rates was consistent with the experimentally reported values. In other words, the two simulations represented lower-viscosity and higher-viscosity variants of clinically relevant shear-thinning biologics, constructed directly from measured material properties.

Using the same porous geometry, boundary conditions, and inlet-pressure profiles defined in Section 3.1, we simulated the flow of these two Cross-model fluids under two driving conditions: the injection-only baseline pressure profile without superimposed vibration and the sinusoidal inlet-pressure profile with the higher vibration amplitude used in this study ($\Delta P_{2}$). For each combination of material properties and driving condition (baseline or high-amplitude vibration $\Delta P_{2}$), we evaluated the time evolution of the wetted area within the porous media, the cycle-integrated pressure per unit wetted area, and the wall-shear-stress history.

**Figure 9:**
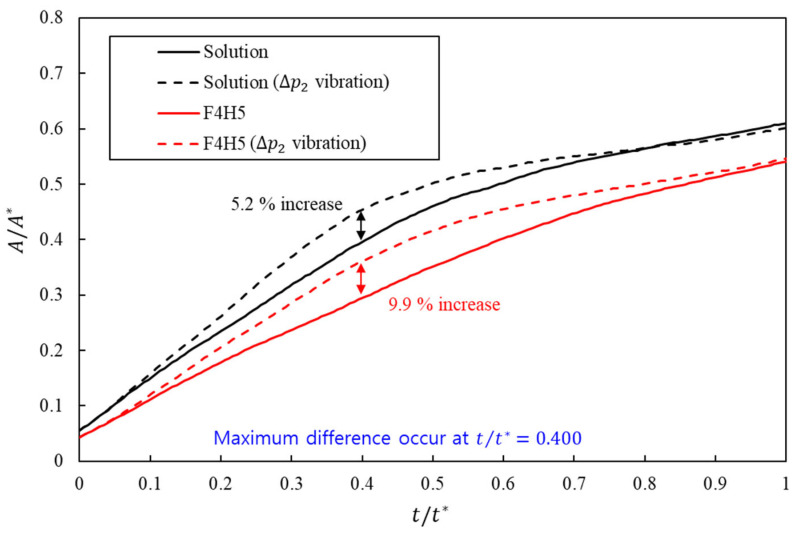
Time evolution of normalized wetting area $A/A^{*}$ versus nondimensional time $t/t^{*}$ for solution and F4H5 with and without inlet-pressure vibration. The black and red colors represent the results for the solution and F4H5, respectively. The dashed line indicates that inlet-pressure vibration was applied.

For the wetted area within the skin surrogate, clinically motivated formulations showed that superimposed inlet-pressure vibration systematically enlarged the effective contact region, with a stronger relative benefit at higher viscosity. In the lower-viscosity solution case, the high-amplitude sinusoidal profile increased the normalized wetting area from 0.409 to 0.430, corresponding to an improvement of approximately 5.2% relative to the injection-only baseline, as shown in Figure 9.

The higher-viscosity F4H5 formulation started from a substantially smaller wetted area under the baseline profile, with a cycle-averaged value of 0.331, which was about 19% lower than that of the solution. When the same high-amplitude vibration was applied, the normalized wetting area in F4H5 increased to 0.364, a gain of approximately 9.9% relative to its own baseline. Vibration therefore partly compensated for the penalty imposed by the higher apparent viscosity.

Together, these results indicate that inlet-pressure vibration enhances wetting for both formulations, with a more pronounced effect in the higher-viscosity F4H5 case. Within the clinically measured viscosity range considered here, the more viscous formulation showed a larger relative increase in wetted area when vibration was applied, indicating that vibration-assisted injection is particularly beneficial for higher-viscosity shear-thinning biologics.

**Figure 10:**
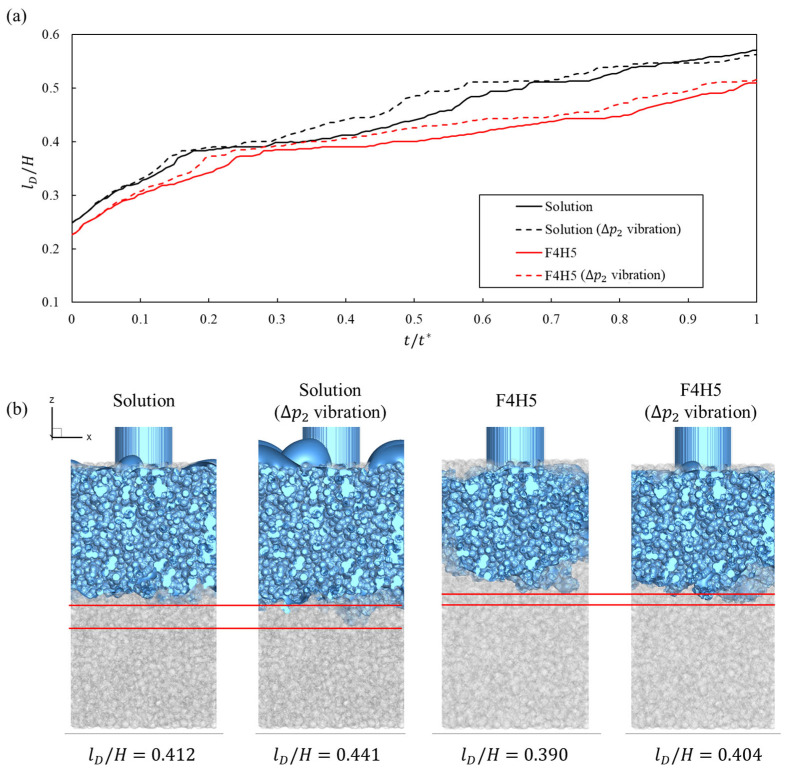
(**a**) Quantitative plot of fluid dispersion length and (**b**) qualitative plot of fluid dispersion length for each fluid type and vibration amplitude at $t/t^{*}=0.400$. The red line has been added to help compare dispersion lengths.

For the clinically measured shear-thinning formulations, Figure 10 shows that these wetting gains are accompanied by consistently larger dispersion lengths under inlet-pressure vibration. As shown in Figure 10a, both the solution and F4H5 exhibited longer normalized dispersion lengths when vibration was applied at the same nondimensional time, with the relative extension being more pronounced for the higher-viscosity F4H5 suspension. The qualitative maps in Figure 10b confirm that vibration not only advanced the penetration front but also broadened the laterally spread region within the porous window, partly compensating for the slower baseline dispersion in F4H5 and reinforcing that oscillatory forcing promotes deeper and wider depot formation in clinically relevant shear-thinning biologics.

For the clinically measured formulations, both of which are shear-thinning, inlet-pressure vibration altered the wall-shear-stress history in a similar way across viscosity but with slightly different magnitudes (Table 5). Under the injection-only baseline profile, the cycle-averaged wall shear stress was 203.43 Pa for the solution and 192.13 Pa for the more viscous F4H5 case, and the late-phase mean was 172.6 and 153.58 Pa, respectively. When the high-amplitude vibration ($\Delta P_{2}$) was applied, the cycle mean decreased only modestly (by about 1.6% in the solution and 0.7% in F4H5), whereas the late-phase mean dropped much more strongly, from 172.6 to 152.59 Pa in the solution and from 153.58 to 136.16 Pa in F4H5, corresponding to reductions of approximately 11.6% and 11.3%. At the same time, the peak wall shear stress increased from 260.36 to 281.19 Pa in the solution and from 255.74 to 269.45 Pa in F4H5 (about 8.0% and 5.4% increases), with the peaks remaining confined to the early part of the cycle in all cases and accompanied by similar increases in the 95th percentile values.

These trends show that, for both viscosities, the high-amplitude vibration did not simply lower shear everywhere but rebalanced the “shear budget” toward short, early-cycle peaks while unloading the interface during the latter part of the injection. Because both the low-viscosity solution and the higher-viscosity F4H5 experienced a comparable (11~12%) reduction in late-phase wall shear, the nociceptor-relevant unloading effect of vibration was preserved across the viscosity range considered here. In combination with the wetting-area results, this indicates that increasing viscosity within a clinically realistic shear-thinning class changes the absolute WSS levels but does not remove the key qualitative benefit of inlet-pressure vibration, which continues to trade a small increase in brief early peaks for a substantial reduction in sustained late-phase shear.

**Figure 11:**
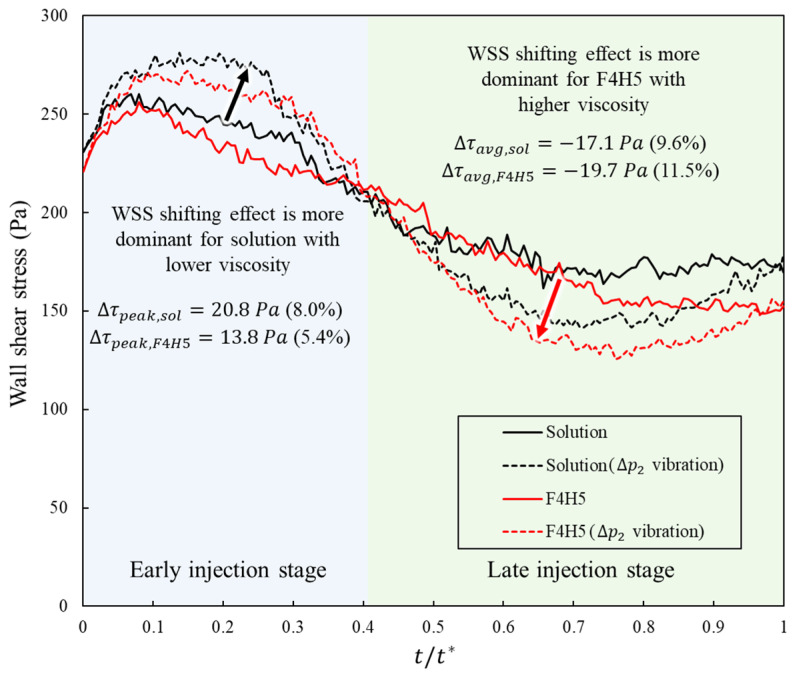
Temporal evolution of wall shear stress for clinically measured shear-thinning protein formulations, separated into an early injection stage ($t/t^{*}\;<\;0.4$, blue background) and a late injection stage ($t/t^{*}\;\geq \;$0.4, green background). Solid lines show the injection-only baseline profile for the solution and F4H5 formulations, and dashed lines show the high-amplitude sinusoidal vibration profile ($\Delta P_{2}$) for each formulation.

Interestingly, as shown in Figure 11, the influence of vibration depends not only on the viscosity level but also on the phase of the injection. In the early part of the cycle, the low-viscosity solution showed the larger relative increase in peak and high percentile wall shear under the high amplitude profile, whereas in the late part of the cycle, the F4H5 formulation exhibited the stronger reduction in mean wall shear with vibration. This behavior was consistent with the interplay between shear-thinning and viscous damping. Early in the injection, shear rates near the inlet were high and both fluids operated close to their high shear viscosity, so the absolute viscosity contrast between 5.1 and 9.2 mPa·s was modest. Under these conditions, the lower-viscosity solution responded more directly to the oscillatory pressure forcing, leading to larger relative perturbations of near-wall velocity and a more pronounced increase in instantaneous shear. As the injection proceeded and shear rates decreased, the effective viscosity of the shear-thinning F4H5 formulation remained higher than that of the solution and the flow field became more diffusion controlled. Without vibration, this higher viscosity sustained a relatively elevated late phase shear at the interface. When vibration was added, the repeated pressure oscillations promoted deeper penetration and lateral spreading, which broadened the shear bearing region and relieved the local near wall gradients more efficiently in the F4H5 case. As a result, the late phase unloading of wall shear was slightly stronger for F4H5, even though the early peaks increased less than in the solution.

**Figure 12:**
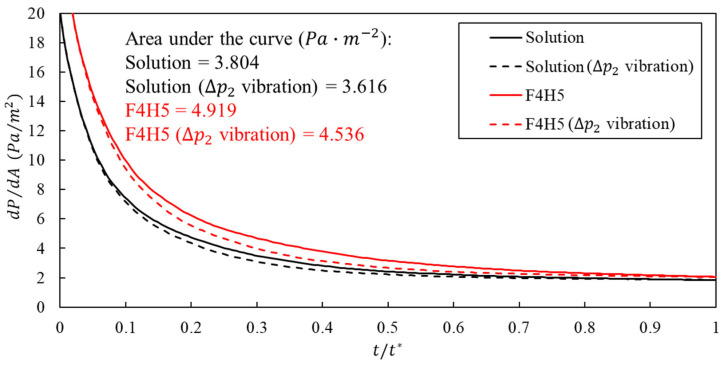
Pressure per wetted area for clinically measured shear-thinning protein formulations. The curves show the pressure per unit wetted area, dP/dA, as a function of normalized time $t/t^{*}$ for the solution (black) and the F4H5 formulation (red) under the injection-only baseline profile (solid lines) and the high-amplitude sinusoidal vibration profile $\Delta P_{2}$ (dashed lines). The area under each curve gives the cycle integrated pressure per unit wetted area.

Figure 12 confirms that higher viscosity increases the pressure cost per wetted area while inlet pressure vibration partly compensates for this penalty. At any given time, dP/dA is larger for the F4H5 formulation than for the solution, consistent with its higher zero shear viscosity. Superimposing the high amplitude sinusoidal component shifts both curves downward over most of the injection so that the cycle integrated pressure per wetted area is reduced by approximately 4.9% for the solution case and by about 7.8% for F4H5. Combined with the wetted area results, these data show that vibration-assisted injection not only enlarges the contacted region but also reduces the pressure spent per unit wetted area, and that this efficiency gain is more pronounced for the higher-viscosity F4H5 formulation within the clinically measured range considered here.

Figure 12 thus also has a clear nociceptive interpretation. In the clinically measured shear-thinning formulations, vibration simultaneously lowers the late-phase mean wall shear stress and the cycle-integrated pressure per wetted area by on the order of 10%, even though the wetted region becomes larger. In subcutaneous and fascial tissue, where nerve endings are sparsely distributed on an areal basis, the mechanically relevant quantity for nociceptor activation is the sustained shear and pressure experienced per receptor rather than the absolute contact area; viewed in this way, the combined WSS and dP/dA trends indicate that vibration-assisted injection is expected to decrease the mechanical drive applied to individual nociceptors while preserving, and in some cases enhancing, the spread of the injected bolus.

## 4. Discussion

In this study, we examined how inlet pressure vibration reorganizes injection flow in a porous, skin-like medium and how this reorganization relates to pain-relevant mechanics and drug delivery. Across Newtonian, model shear-thinning, and clinically measured protein formulations, vibration enlarged the contacted region, advanced the penetration front, and increased dispersion within the matrix while reducing the pressure required per unit area of contact. These trends indicate that clinically realistic vibration amplitudes can improve depot formation without changing formulation composition or injection volume.

A key finding is that vibration does not simply add mechanical loading on top of baseline injection but redistributes it in time. For shear-thinning fluids, the wall-shear-stress history was front loaded, with a modestly amplified early peak and a consistent reduction in late-phase mean values on the order of ten percent. At the same time, the pressure cost per wetted area decreased while wetting and the dispersion length increased. Interpreted together, these results suggest that vibration can reduce sustained wall shear and pressure that drive nociceptor activation while expanding the exchange surface that supports transport and uptake.

The case study with clinically measured high concentration protein formulations showed that these mechanisms persist for drug-like systems with substantially different viscosities. Both the lower-viscosity solution and the more viscous suspension benefited from vibration, with relatively larger gains in depot size and pressure efficiency for the higher-viscosity case. This pattern implies that device level vibration may be particularly useful for challenging, high-viscosity formulations that are otherwise associated with high injection forces and poor tolerability. In practical terms, vibration amplitude and injectate rheology emerge as coupled design variables that can be tuned to balance comfort and delivery performance.

Several limitations should be acknowledged. The tissue was modeled as a rigid, homogeneous porous medium with an idealized window around a fixed needle, so deformation, structural heterogeneity, and two-way poromechanical coupling were not represented. Only one geometry, one vibration frequency, and a limited set of amplitudes were considered, and the non-Newtonian behavior was captured with a simplified shear-thinning model that did not include thixotropy or viscoelasticity. Nociception was inferred from mechanical surrogate metrics rather than an explicit neural model. Future work that incorporates compliant and heterogeneous tissue, richer rheology, varied vibration patterns, and anatomically informed nociceptor representations will be needed to translate these fluid mechanical insights into quantitative predictions of pain and to refine vibration-assisted injection protocols for specific devices and formulations.

## Figures and Tables

**Table 1:** Domain information and simulation parameters.

Variables	Symbols	Lattice Values	Physical Values	Units
Length conversion factor	$\delta_{x}$	-	$1.33\times 10^{-6}$	m
Time conversion factor	$\delta_{t}$	-	$1.90\times 10^{-8}$	s
Mass conversion factor	$\delta_{m}$	-	$3.26\times 10^{-16}$	kg
Width of simulation domain	W	240	0.319	mm
Height of simulation domain	H	510	0.678	mm
Needle injection length	$h_{insert}$	150	0.199	mm
Inlet radius	D	90	0.11	mm
Liquid density	$\rho_{l}$	6.6314	913.2	$kg/m^{3}$
Liquid viscosity	$\nu_{l}$	0.7104	9.13	cP
Air density	$\rho_{v}$	0.3417	47.06	$kg/m^{3}$
Air viscosity	$\nu_{v}$	0.1623	2.09	cP

**Table 2:** Properties used for Cross model.

Fluid	$\mu_{0}$ (mPa·s)	$\mu_{\infty }$ (mPa·s)	k (s)	n
Newtonian	2.5	-	-	-
Non-Newtonian 1	2.5	0.25	0.0015	0.8
Non-Newtonian 2	2.5	0.25	0.01	0.65

**Table 3:** Normalized wetting area $A/A^{*}$ and percent improvement at $t/t^{*}=0.434$ relative to the matched no-vibration baseline for each fluid type and vibration amplitude.

Simulation Conditions	$A/A^{*}$	Improvement (%)
Newtonian	0.119	-
Newtonian $(\Delta P_{1}$ vibration)	0.131	10.6
Non-Newtonian	0.315	-
Non-Newtonian $(\Delta P_{1}$ vibration)	0.365	15.9
Non-Newtonian $(\Delta P_{2}$ vibration)	0.382	21.3

**Table 4:** One-cycle summary of wall shear stress (WSS) with pain-relevant metrics. WSS τ is reported in Pascal units over one pressure cycle. $\overset{⃐}{\tau }$: cycle mean. ${\overset{⃐}{\tau }}_{0.4\to 1.0}$: mean over the final 60% of the cycle. $\tau_{95}$: 95th percentile over the cycle. Peak τ: maximum value within the cycle. Peak time: nondimensional time at which the peak occurs.

Simulation Conditions	$\overset{⃐}{\tau }$ **(Pa)**	${\overset{⃐}{\tau }}_{0.4\to 1.0}$ **(Pa)**	Peak τ **(Pa)**	$\tau_{95}$ **(Pa)**	Peak Time
Newtonian	126.82	142.64	151.78	147.08	1.000
Newtonian $(\Delta P_{1}$ vibration)	194.80	162.02	247.24	243.98	0.131
Non-Newtonian	195.33	158.62	256.23	249.86	0.080
Non-Newtonian $(\Delta P_{1}$ vibration)	192.74	142.22	267.26	262.98	0.103
Non-Newtonian $(\Delta P_{2}$ vibration)	191.89	137.53	271.90	267.56	0.149

**Table 5:** One-cycle summary of wall shear stress (WSS) with pain-relevant metrics for protein formulations. WSS τ is reported in Pascal units over one pressure cycle. $\overset{⃐}{\tau }$: cycle mean. ${\overset{⃐}{\tau }}_{0.4\to 1.0}$: mean over the final 60% of the cycle. $\tau_{95}$: 95th percentile over the cycle. Peak τ: maximum value within the cycle. Peak time: nondimensional time at which the peak occurs.

Simulation Conditions	$\overset{⃐}{\tau }$ **(Pa)**	${\overset{⃐}{\tau }}_{0.4\to 1.0}$ **(Pa)**	Peak τ **(Pa)**	$\tau_{95}$ **(Pa)**	Peak Time
Solution	203.43	172.6	260.36	254.85	0.069
Solution $(\Delta P_{2}$ vibration)	200.16	152.59	281.19	277.23	0.137
F4H5	192.13	153.58	255.74	247.26	0.083
F4H5 $(\Delta P_{2}$ vibration)	190.76	136.16	269.45	264.49	0.152

## Data Availability

The raw data supporting the conclusions of this article will be made available by the authors on request.

## References

[1] McLenon J., Rogers M.A. (2019). The fear of needles: A systematic review and meta-analysis. J. Adv. Nurs..

[2] Alsbrooks K., Hoerauf K. (2022). Prevalence, causes, impacts, and management of needle phobia: An international survey of a general adult population. PLoS ONE.

[3] McMurtry C.M., Riddell R.P., Taddio A., Racine N., Asmundson G.J., Noel M., Chambers C.T., Shah V., HELPinKids&Adults Team (2015). Far from “just a poke”: Common painful needle procedures and the development of needle fear. Clin. J. Pain.

[4] Taddio A., Appleton M., Bortolussi R., Chambers C., Dubey V., Halperin S., Hanrahan A., Ipp M., Lockett D., MacDonald N. (2010). Reducing the pain of childhood vaccination: An evidence-based clinical practice guideline. Can. Med. Assoc. J..

[5] Taddio A., Ipp M., Thivakaran S., Jamal A., Parikh C., Smart S., Sovran J., Stephens D., Katz J. (2012). Survey of the prevalence of immunization non-compliance due to needle fears in children and adults. Vaccine.

[6] Comite S.L., Rahaman S., Malkowiak M. (2024). Vibration Anesthesia During Invasive Procedures: A Meta-analysis. J. Clin. Aesthetic Dermatol..

[7] Fix W.C., Chiesa-Fuxench Z.C., Shin T., Etzkorn J., Howe N., Miller C.J., Sobanko J.F. (2019). Use of a vibrating kinetic anesthesia device reduces the pain of lidocaine injections: A randomized split-body trial. J. Am. Acad. Dermatol..

[8] Kazi R., Govas P., Slaugenhaupt R.M., Carroll B.T. (2020). Differential analgesia from vibratory stimulation during local injection of anesthetic: A randomized clinical trial. Dermatol. Surg..

[9] Mortada H., Al Qurashi A.A., Alnaim M.F., Arab K., Kattan A.E. (2024). Effectiveness of using a vibration device to ease pain during upper extremity injections: A randomized controlled trial. Saudi J. Anaesth..

[10] Clement R.S., Unger E.L., Ocón-Grove O.M., Cronin T.L., Mulvihill M.L. (2016). Effects of axial vibration on needle insertion into the tail veins of rats and subsequent serial blood corticosterone levels. J. Am. Assoc. Lab. Anim. Sci..

[11] Gidde S.T.R., Ciuciu A., Devaravar N., Doracio R., Kianzad K., Hutapea P. (2020). Effect of vibration on insertion force and deflection of bioinspired needle in tissues. Bioinspiration Biomim..

[12] Perra E., Lampsijärvi E., Barreto G., Arif M., Puranen T., Hæggström E., Pritzker K.P., Nieminen H.J. (2021). Ultrasonic actuation of a fine-needle improves biopsy yield. Sci. Rep..

[13] Marathe D., Bhuvanashree V.S., Mehta C.H., T A., Nayak U.Y. (2024). Low-Frequency Sonophoresis: A Promising Strategy for Enhanced Transdermal Delivery. Adv. Pharmacol. Pharm. Sci..

[14] Smith F., Kotowska A.M., Fiedler B., Cerny E., Cheung K., Rutland C.S., Chowdhury F., Segal J., Rawson F.J., Marlow M. (2024). Using Oscillation to Improve the Insertion Depth and Consistency of Hollow Microneedles for Transdermal Insulin Delivery with Mechanistic Insights. Mol. Pharm..

[15] Kalisman D., Yakirevich A., Sorek S., Kamai T. (2023). Impact of pressure waves on water imbibition and flow in unsaturated porous media. Water Resour. Res..

[16] Xiao M., Reddi L.N., Steinberg S.L. (2006). Effect of vibrations on pore fluid distribution in porous media. Transp. Porous Media.

[17] Chernos M., Grecov D., Kwok E., Bebe S., Babsola O., Anastassiades T. (2017). Rheological study of hyaluronic acid derivatives. Biomed. Eng. Lett..

[18] Fundarò S.P., Salti G., Malgapo D.M.H., Innocenti S. (2022). The rheology and physicochemical characteristics of hyaluronic acid fillers: Their clinical implications. Int. J. Mol. Sci..

[19] Hong G.-W., Wan J., Park Y., Chang K., Chan L.K.W., Lee K.W.A., Yi K.-H. (2024). Rheological characteristics of hyaluronic acid fillers as viscoelastic substances. Polymers.

[20] Rebenda D., Vrbka M., Čípek P., Toropitsyn E., Nečas D., Pravda M., Hartl M. (2020). On the dependence of rheology of hyaluronic acid solutions and frictional behavior of articular cartilage. Materials.

[21] Gupta J., Park S.S., Bondy B., Felner E.I., Prausnitz M.R. (2011). Infusion pressure and pain during microneedle injection into skin of human subjects. Biomaterials.

[22] Liu H., Kang Q., Leonardi C.R., Schmieschek S., Narváez A., Jones B.D., Williams J.R., Valocchi A.J., Harting J. (2016). Multiphase lattice Boltzmann simulations for porous media applications: A review. Comput. Geosci..

[23] Kupershtokh A.L., Medvedev D., Karpov D. (2009). On equations of state in a lattice Boltzmann method. Comput. Math. Appl..

[24] Gong S., Cheng P. (2012). Numerical investigation of droplet motion and coalescence by an improved lattice Boltzmann model for phase transitions and multiphase flows. Comput. Fluids.

[25] Sohrabi S., Liu Y. (2018). Modeling thermal inkjet and cell printing process using modified pseudopotential and thermal lattice Boltzmann methods. Phys. Rev. E.

[26] Bird R.B., Armstrong R.C., Hassager O. (1987). Fluid mechanics. Dynamics of Polymeric Liquids.

[27] Miller P.R., Taylor R.M., Tran B.Q., Boyd G., Glaros T., Chavez V.H., Krishnakumar R., Sinha A., Poorey K., Williams K.P. (2018). Extraction and biomolecular analysis of dermal interstitial fluid collected with hollow microneedles. Commun. Biol..

[28] Gradel A.K.J., Porsgaard T., Lykkesfeldt J., Seested T., Gram-Nielsen S., Kristensen N.R., Refsgaard H.H.F. (2018). Factors affecting the absorption of subcutaneously administered insulin: Effect on variability. J. Diabetes Res..

[29] Pertinez H., Kaushik A., Curley P., Arshad U., El-Khateeb E., Li S.-Y., Tasneen R., Sharp J., Kijak E., Herriott J. (2024). Hyaluronidase impacts exposures of long-acting injectable paliperidone palmitate in rodent models. bioRxiv.

[30] Thomas J.R., Wallace M.S., Yocum R.C., Vaughn D.E., Haller M.F., Flament J. (2009). The INFUSE-Morphine study: Use of recombinant human hyaluronidase (rHuPH20) to enhance the absorption of subcutaneously administered morphine in patients with advanced illness. J. Pain Symptom Manag..

[31] Woodley W.D., Morel D.R., Sutter D.E., Pettis R.J., Bolick N.G. (2022). Clinical evaluation of large volume subcutaneous injection tissue effects, pain, and acceptability in healthy adults. Clin. Transl. Sci..

[32] Pettis R.J., Muchmore D., Heinemann L. (2019). Subcutaneous insulin administration: Sufficient progress or ongoing need?. J. Diabetes Sci. Technol..

[33] Pettis R.J., Woodley W.D., Ossege K.C., Blum A., Bolick N.G., Rini C.J. (2023). Imaging of large volume subcutaneous deposition using MRI: Exploratory clinical study results. Drug Deliv. Transl. Res..

[34] de Lucio M., Leng Y., Wang H., Vlachos P.P., Gomez H. (2024). Modeling drug transport and absorption in subcutaneous injection of monoclonal antibodies: Impact of tissue deformation, devices, and physiology. Int. J. Pharm..

[35] Wang H., Hu T., Leng Y., de Lucio M., Gomez H. (2023). MPET2: A multi-network poroelastic and transport theory for predicting absorption of monoclonal antibodies delivered by subcutaneous injection. Drug Deliv..

[36] Gong J., Chen J., Gu P., Shang Y., Ruppell K.T., Yang Y., Wang F., Wen Q., Xiang Y. (2022). Shear stress activates nociceptors to drive Drosophila mechanical nociception. Neuron.

[37] Marschall C., Witt M., Hauptmeier B., Frieß W. (2023). Drug product characterization of high concentration non-aqueous protein powder suspensions. J. Pharm. Sci..

